# The prevalence of Post-Ebola Syndrome hearing loss, Sierra Leone

**DOI:** 10.1186/s12879-022-07604-y

**Published:** 2022-07-18

**Authors:** Samuel C. Ficenec, Donald S. Grant, Ibrahim Sumah, Foday Alhasan, Mohamed S. Yillah, Jenneh Brima, Edwin Konuwa, Michael A. Gbakie, Fatima K. Kamara, Nell G. Bond, Emily J. Engel, Jeffrey G. Shaffer, William A. Fischer, David A. Wohl, Susan D. Emmett, John S. Schieffelin

**Affiliations:** 1 Department of Internal Medicine, Tulane University School of Medicine, New Orleans, LA USA; 2 Kenema Government Hospital, Ministry of Health and Sanitation, Kenema, Sierra Leone; 3 College of Medicine and Allied Health Sciences, University of Sierra Leone, Freetown, Sierra Leone; 4 Department of Immunology and Microbiology, Tulane University School of Medicine, New Orleans, LA USA; 5 Department of Pediatrics, Section of Infectious Diseases, Tulane University School of Medicine, New Orleans, LA USA; 6 Department of Biostatistics and Data Science, Tulane University School of Public Health and Tropical Medicine, New Orleans, LA USA; 7 Department of Internal Medicine, Division of Pulmonary Diseases and Critical Care Medicine, University of North Carolina School of Medicine, Chapel Hill, NC USA; 8 Department of Internal Medicine, Division of Infectious Diseases, University of North Carolina School of Medicine, Chapel Hill, NC USA; 9 Department of Epidemiology, University of Arkansas for Medical Sciences, Little Rock, AR USA; 10 Department of Otolaryngology, University of Arkansas for Medical Sciences, Little Rock, AR USA

## Abstract

**Background:**

Globally, hearing loss is the second leading cause of disability, affecting approximately 18.7% of the world’s population. However, the burden of hearing loss is unequally distributed, with the majority of affected individuals located in Asia or Sub-Saharan Africa. Following the 2014 West African Ebola Outbreak, disease survivors began to describe hearing loss as part of the constellation of symptoms known as Post-Ebola Syndrome. The goal of this study was to more fully characterize hearing loss among Ebola Virus Disease (EVD) survivors.

**Methodology and principal findings:**

EVD survivors and their household contacts were recruited (n = 1,12) from Eastern Sierra Leone. Each individual completed a symptom questionnaire, physical exam, and a two-step audiometry process measuring both air and bone conduction thresholds. In comparison to contacts, EVD survivors were more likely to have complaints or abnormal findings affecting every organ system. A significantly greater percentage of EVD survivors were found to have hearing loss in comparison to contacts (23% vs. 9%, p < 0.001). Additionally, survivors were more likely to have bilateral hearing loss of a mixed etiology. Logistic regression revealed that the presence of any symptoms of middle or inner ear (p < 0.001), eye (p = 0.005), psychiatric (p = 0.019), and nervous system (p = 0.037) increased the odds of developing hearing loss.

**Conclusions and significance:**

This study is the first to use an objective and standardized measurement to report hearing loss among EVD survivors in a clinically meaningful manner. In this study it was found that greater than 1/5th of EVD survivors develop hearing loss. The association between hearing impairment and symptoms affecting the eye and nervous system may indicate a similar mechanism of pathogenesis, which should be investigated further. Due to the quality of life and socioeconomic detriments associated with untreated hearing loss, a greater emphasis must be placed on understanding and mitigating hearing loss following survival to aid in economic recovery following infectious disease epidemics.

**Supplementary Information:**

The online version contains supplementary material available at 10.1186/s12879-022-07604-y.

## Background

Data from the global burden of disease study indicates that hearing loss is currently the second leading cause of disability-adjusted life years (DALYs) [1]. Approximately 18.7% of the world’s population or 1.5 billion people live with hearing loss, and this number is expected to increase throughout the following decades [1–4]. If left untreated, hearing loss can cause substantial detriments to an individual’s quality of life and socioeconomic capacity. This disability increases the risk of being under- or unemployed, impairs the completion of many activities of daily living, and increases the likelihood of social isolation and depression in adults. Furthermore, children with untreated hearing loss are more likely to experience speech and language delays, poor school performance, and increased likelihood of dropout [5–11]. Globally, the burden of hearing loss is unequally distributed, with over 80% of individuals living within low- and middle-income countries (LMIC) where accessibility to treatment is limited [2, 12, 13]. In order to mitigate this health disparity, a more complete understanding of the etiologies of hearing loss in LMICs is needed.

It is well known that individuals living in these environments suffer from higher rates of bacterial and viral meningitis and the neurologic sequelae associated with these infections [14, 15]. Hearing loss secondary to these infections is thought to occur due to overactivation of the immune system and direct toxicity causing damage to the cochlea [16–18]. However, the pathophysiology of hearing loss related to lesser understood tropical viruses remains poorly characterized.

Ebola virus has caused several large epidemics throughout Sub-Saharan Africa costing thousands of lives [19, 20]. The West African Ebola Outbreak of 2013 to 2016, which began in Guinea in December 2013 remains the largest Ebola outbreak to date [21]. The virus quickly spread throughout West Africa infecting over 28,000 individuals and claiming the lives of over 11,000 [22]. As Ebola Virus Disease (EVD) patients continued to reach convalescence, a number of symptoms including musculoskeletal pain, ocular complaints, and hearing loss were noted among survivors [23–26]. This constellation of symptoms which has been shown to arise in over 90% of survivors was termed Post-Ebola Syndrome [25, 27]. Previous data has indicated that hearing loss occurs in approximately 0–64% of survivors [28–32]. However, due to lack of standardized reporting and objective measurement of hearing thresholds, comparison of any study of Post-Ebola Syndrome hearing loss is difficult [33].

The case control study described here sought to characterize Post-Ebola Syndrome hearing loss. Characterization of this and other sequelae may help elucidate the pathogenesis of Post-Ebola Syndrome, identify treatment and intervention options, and increase understanding of the full disease course of EVD. An increased understanding of this disease course will allow for improved ability to design interventions and treatments to mitigate and prevent the acute and chronic symptoms of EVD affecting these tropical and LMIC.

## Methods

### Study design

This study was conducted in eastern Sierra Leone from July 2018 to June 2019. Most participants were already enrolled in an ongoing longitudinal study of EVD survivors and household contacts in Sierra Leone [32]. EVD survivors of the West African Ebola outbreak were eligible for enrollment if they were seven years of age or older, if they were registered with the Sierra Leone Association of EVD survivors, and lived in the Eastern Province of Sierra Leone. Each EVD survivor was asked to recruit up to three additional individuals without a history of EBOV infection from their household or village. These additional individuals serve as the control population for the cohort of EVD survivors.

### Symptom questionnaire and physical exams

All individuals completed an extensive questionnaire concerning constitutional, psychiatric, neurologic, ocular, audiovestibular, respiratory, cardiac, gastrointestinal, urorenal, and reproductive or sexual symptoms experienced at the time of the audiometry testing. Additionally, all individuals were given a full physical exam completed by a government hospital physician and a two-step audiometry process.

### Audiometry exams

The first step of the audiometry exam employed the use of a portable Ambco 650AB audiometer (Tustin, CA). Pure tone air conduction thresholds were measured from 0.25 to 8 kHz and pure tone averages (PTA) were calculated using 0.5, 1, 2, and 4 kHz thresholds for each ear. If individuals were found to have a threshold ≥ 25 dB in at least one ear at one or more frequencies, they were referred to the second confirmatory step of the audiometry process. The second step of audiometry testing measured pure tone air and bone conduction thresholds using the SHOEBOX® Audiometry Pro Edition (Ottawa, ON, CA). PTAs were then calculated to determine the presence of any hearing loss [34]. At the time of the study hearing loss was defined as the current WHO standards: mild hearing loss (PTA > 25 and ≤ 40 dB), moderate hearing loss (PTA > 40 dB), severe hearing loss (PTA > 60 dB), and profound hearing loss (PTA > 80 dB) [35]. In this study, hearing loss type was determined based on an air bone gap of 10 dB (a measured difference of 10 dB between air and bone conduction thresholds). Individuals with hearing loss without a 10 dB air bone gap across any hearing threshold were deemed to have pure sensorineural hearing loss. Those with at least one 10 dB air bone gap were deemed to have mixed hearing loss. Individuals with hearing loss with a 10 dB or greater air bone gap across every threshold were deemed to have pure conductive hearing loss.

### Statistical analysis

Data was collected on standardized forms, entered into Microsoft Access (Redmond, WA) databases, imported into R statistical software, and manipulated within the RStudio environment [36, 37]. Significant differences between EVD survivors and contacts were found through the use of the Student’s t test for ordinal or interval level data and Pearson’s chi-squared and Fisher’s exact tests for nominal variables where appropriate. In instances where this was not true logistic regression modelling was done to control for known confounding variables. These statistical tests were loaded from the R stats and base packages, respectively [36]. To fully characterize the differences among EVD survivors and contacts, audiometry data for each survivor was sorted and assessed based on the ear with the highest PTA. Data on symptoms and physical exam findings were then grouped into aggregate variables by the affected organ system. The symptoms and exam maneuvers to assess the middle and inner ear include: dizziness, hearing loss, tinnitus, ear fullness, and audiometric exams. Other organ system data is defined in Additional file 1: Tables S1 and S2). Normal values for heart rate, respiratory rate, and blood pressure vary significantly by age for children under 15 years and were analyzed separately. Vital signs were grouped into Systemic Physical exam findings. Logistic regression modelling using a forward and step-wise methodology was performed on the aggregate variables, using the glm function loaded from the stats package in R to assess their association with development of hearing loss. As hearing loss in this region is most commonly caused by otitis media and bacterial pathogens, hearing loss in this model was restricted to sensorineural and mixed etiologies in order to control for confounding effects [13, 38]. All aggregate variables were assessed for inclusion in the model, variables were added to the model if significant improvements were made to the fit of the model based on the likelihood-ratio test. A classifier decision tree was created to determine non-linear relationships between aggregate variables and the development of sensorineural or mixed etiology hearing loss using the train and train control functions loaded from the caret package in R. The gini impurity index was used as the criterion to determine splits in the data, the model was created using 70% (n = 810) of the EVD cohort and 10 times cross-validation. This model was tested on the remaining 30% (n = 202) of the cohort that was not used to create the original decision tree model. The accuracy of the decision tree model was compared to the no information rate as standard methodology for testing the significance of classification models. The no information rate is defined as the error rate that would be achieved by classifying every subject as the majority class label. Effects of known confounding variables such as age and sex were controlled for by inclusion in statistical tests, logistic regression, and decision tree models where applicable. The accompanying figures included in this manuscript were created using software produced by Prism (San Diego, CA), R Core Team (Vienna, Austria), and Microsoft (Redmond, WA). In the instance that data was found to be missing for a single value it was imputed to be negative or zero.

## Results

**Fig. 1:**
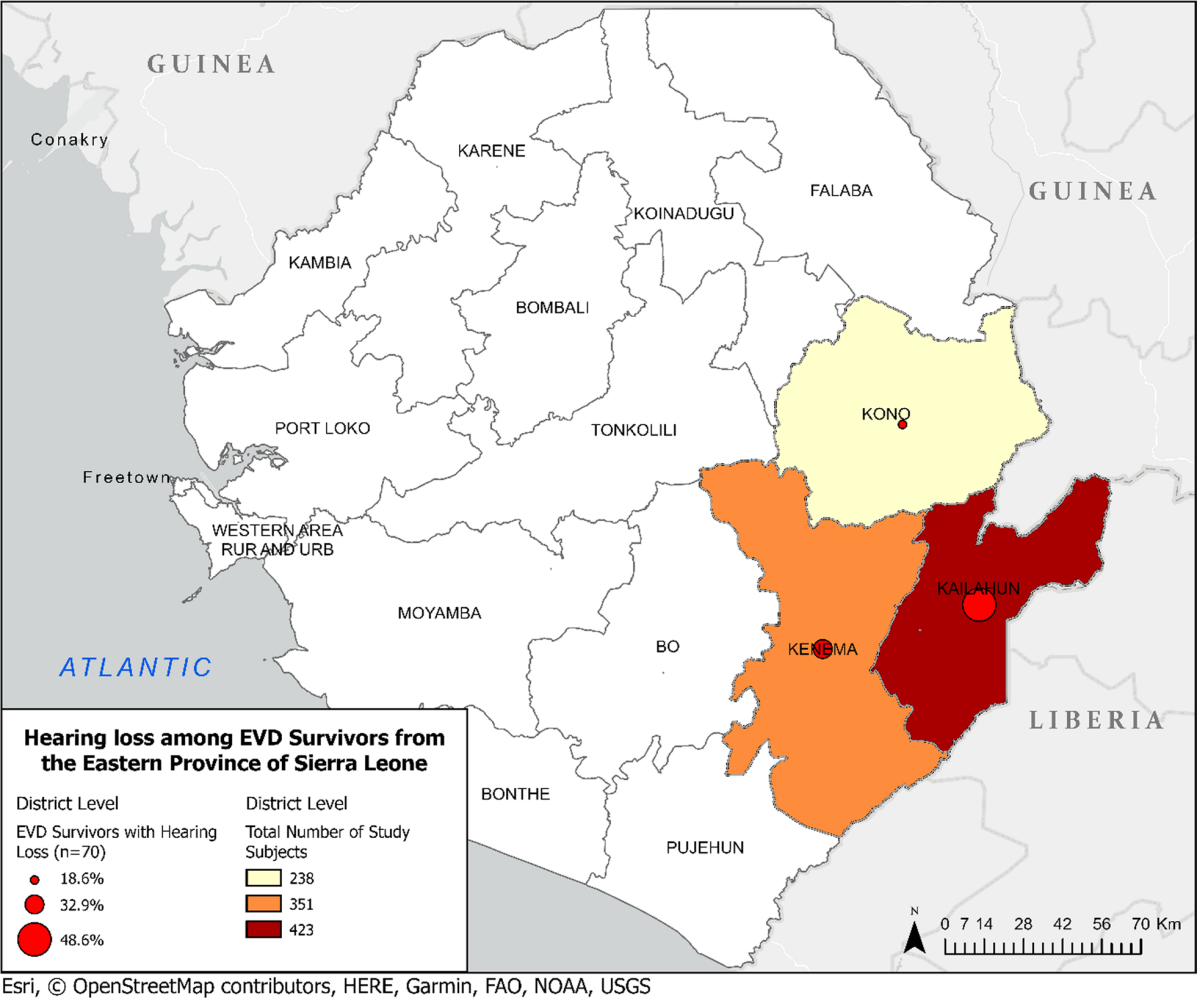
Geographic distribution of participants and associated hearing loss (HL) among EVD survivors. Map depicting distribution of enrolled participants across Kailahun, Kenema, and Kono Districts. To investigate for potential location dependent effects of hearing loss, the proportion of EVD survivors from each District was noted and is represented by the red circle and their relative size. No significant differences were noted among EVD survivors across District level (p = 0.900)

By the end of the study period in May 2019, a total of 1,012 individuals were enrolled into the study cohort and completed at least one study visit including audiometry testing. All subjects enrolled in this study were recruited from the Kailahun, Kenema, and Kono Districts in Eastern Sierra Leone (Fig. 1). The majority of study participants were recruited from Kailahun (n = 423, 42%) District followed by Kenema (n = 351, 35%), and Kono (n = 238, 23%) Districts. The total study cohort consisted of 301 EVD survivors and 711 contacts (Table 1). The mean time from EVD diagnosis to audiometry testing was 4.3 years. EVD survivors were significantly older and more likely to be female in comparison to contacts.

**Table 1:** Participant demographics and vital signs

	EVD survivors (n = 301)	EVD contacts (n = 711)	p-value^*^
Mean Age ± Std Dev (Years)	30.2 ± 14.9	22.4 ± 12.0	< 0.001^†^
Females (%)	171 (57)	334 (47)	0.004
Individuals under 15 (%)	50 (17)	181 (25)	0.003
Mean Time since diagnosis (Yrs ± SD)	4.3 ± 0.4	-	-
Mean temperature (°C)^‡^	37	37	0.056^†^
Heart rate (BPM)^‡^	83	82	0.572
Respiratory rate (RPM)^‡^	19	20	0.205
Systolic blood pressure (mm Hg)^‡^	124	122	0.237
Diastolic blood pressure (mm Hg)^‡^	79	76	< 0.001
Mean arterial pressure (mm Hg)^‡^	94	91	0.002
Oxygen saturation	96	98	0.502^†^
BMI	24	22	0.014^†^

Std Dev: Standard deviation; BPM: beats per minute; RPM: respirations per minute; *Pearson’s Chi-Squared Test ; ^†^Student’s T Test; Test; ^‡^Mean values displayed only include individuals ≥ 15 years of age due to age associated variability

Symptom questionnaires revealed that EVD survivors experienced significantly more complaints at the time of visit than household contacts (Additonal file 1: Table S1). Survivors were significantly more likely to complain of 43 out of 53 (81%) symptoms than household contacts. Although all vital signs were within normal limits for both groups, adult (greater than age 15) EVD survivors were found to have a significantly greater BMI, mean arterial pressure, and diastolic blood pressure. Pediatric EVD survivors (less than 15 years of age) were found to have a significantly greater oxygen saturation and respiratory rate. However, none of the differences among vital signs are clinically significant. Individual physical exam maneuvers revealed several significant differences between EVD survivors and household contacts (Additional file 1: Table S2). EVD survivors were more likely than household contacts to have an abnormal pupillary response to light (4%, vs. 2%, p = 0.025), abdominal tenderness (4% vs. 2%, p = 0.015), hepatomegaly (3% vs. < 1%, p = 0.001), splenomegaly (16% vs. 7%, p < 0.001), decreased range of motion of any joint (4% vs. 1%, p = 0.006), and at least one joint tender to palpation (4% vs. 0%, p < 0.001).

**Fig. 2:**
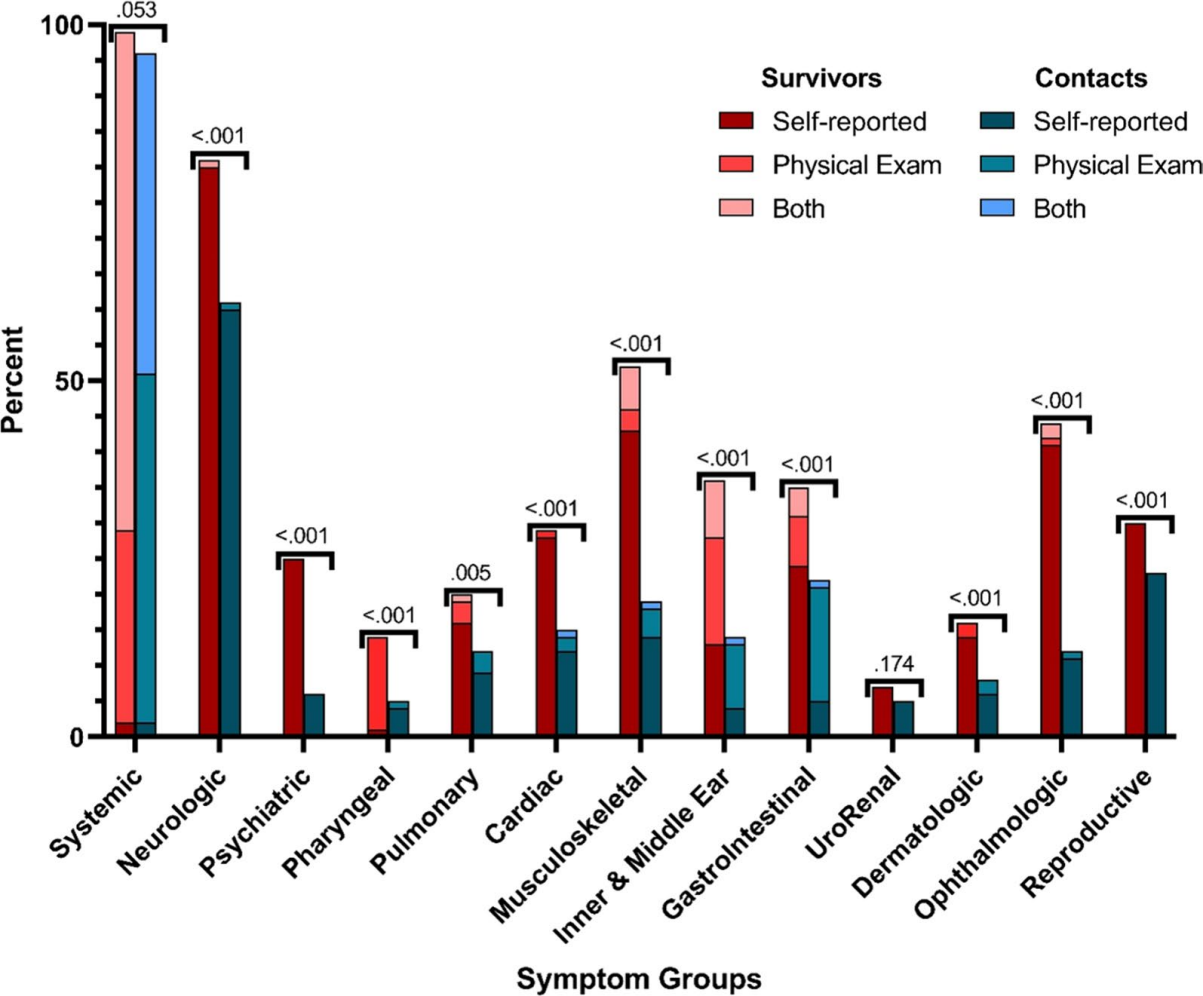
Symptom survey and physical exam organ system findings. Graph displays data gathered through symptom surveys and physical exams. Individual survey questions and physical exam maneuvers were aggregated according to the respective organ system affected

Symptom surveys and physical exams were organized and grouped into aggregate variables according to the affected organ system (Fig. 2). When physical exam and symptom survey data are pooled and viewed together, EVD survivors were more likely to have abnormal findings in every organ system except the systemic and urorenal organ systems. Similar results were found in symptom survey data (Table 2). Interestingly, among physical exam variables, significant differences were only noted among inner and middle ear, gastrointestinal, and ophthalmologic exams (Table 2).

**Table 2:** Symptom survey and physical exam aggregated variable findings

Aggregate variable n (%)	EVD survivors (n = 301)		EVD contacts (n = 711)		p-value^*^	
	Survey	Physical exam	Survey	Physical exam	Survey	Physical exam
Systemic	217 (72)	293 (97)	336 (47)	671 (94)	< 0.001	0.061
Neurologic	244 (81)	2 (1)	430 (60)	7 (1)	< 0.001	0.897
Psychiatric	75 (25)	–	40 (6)	–	< 0.001	-
Pharyngeal	39 (13)	3 (1)	29 (4)	4 (1)	< 0.001	0.729
Pulmonary	50 (17)	10 (3)	69 (10)	20 (3)	0.003	0.815
Cardiac	85 (28)	4 (1)	88 (12)	15 (2)	< 0.001	0.560
Musculoskeletal	147 (49)	26 (9)	106 (15)	37 (5)	< 0.001	0.054
Inner and Middle Ear^†^	65 (22)	70 (23)	33 (5)	67 (9)	< 0.001	< 0.001
Gastrointestinal	84 (28)	33 (11)	120 (17)	47 (7)	< 0.001	0.027
UroRenal	21 (7)	–	33 (5)	–	0.174	–
Dermatologic	42 (14)	7 (2)	43 (6)	16 (2)	< 0.001	1.000
Ophthalmologic	130 (43)	8 (3)	76 (11)	4 (1)	< 0.001	0.013
Reproductive	90 (30)	–	162 (23)	–	0.021	–

*Pearson’s Chi-Squared test; ^†^Includes measurement of hearing thresholds which are removed in later modelling

A two-step audiometry process was conducted on 1,012 study participants. A total of 319 individuals including 120 (40%) EVD survivors and 199 (28%, p < 0.001) household contacts failed the first step of the audiometric exam and completed additional audiometry testing. One hundred thirty-seven individuals were confirmed to have pure tone averages ≥ 25 dB in at least one ear and met criteria for hearing loss (Table 3). This total includes 70 (23%) EVD survivors and 67 (9%, p < 0.001) household contacts. A trend of greater bilateral hearing loss was noted among EVD survivors, however this finding was not significant. When examining the severity of hearing loss, EVD survors had a greater proportion of individuals in every category. However, after controlling for age and sex, this finding was only significant among those with mild hearing loss (12% vs. 4%, p < 0.001). The location of survivors with hearing loss was recorded to investigate potential location-dependent effects of hearing loss among EVD survivors (Fig. 1). Across the District level, the amount of EVD survivors with hearing loss was proportional to the number of individuals recruited from each district (Fig. 1, p = 0.900).

**Table 3:** EVD cohort hearing loss results

	EVD survivors (n = 301)	EVD contacts (n = 711)	p-value*
Any Hearing Loss n (%)	70 (23)	67 (9)	<0.001
Bilateral Hearing Loss n (%)	44 (63)	31 (46)	0.051

Hearing loss yype			
	EVD survivors (n = 70)	EVD contacts (n = 67)	
Sensorineural n (%)	10 (14)	7 (10)	0.772
Mixed n (%)	44 (63)	35 (52)	
Conductive n (%)	16 (23)	25 (37)	

Hearing loss severity			
	EVD survivors (n = 301)	EVD contacts (n = 711)	
None ≤ 25 dB n (%)	231 (77)	644 (91)	<0.001
Mild > 25 dB n (%)	37 (12)	30 (4)	
Moderate > 40 dB n (%)	20 (7)	26 (4)	
Severe > 60 dB n (%)	7 (2)	7 (1)	
Profound > 80 dB n (%)	6 (2)	4 (1)	

*Statistical analysis comparing survivors to contacts was done using logistic regression in R Studio

**Fig. 3:**
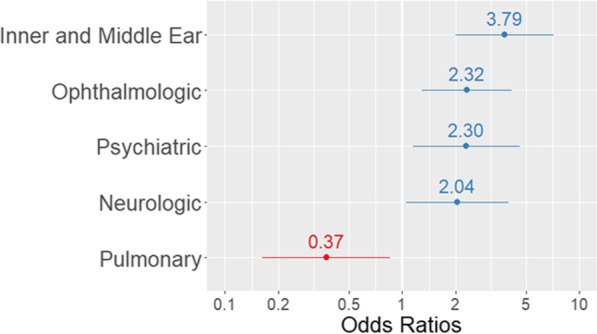
Logistic regression results of sensorineural and mixed hearing loss among EVD survivors. The organ systems listed on the Y axis of regression model were found to significantly affect the odds of developing the most common type of hearing loss associated with EVD. Each point represents the odds of having hearing loss if complaints or abnormal findings were noted affecting each respective organ systems. 95% confidence intervals represented by solid lines

Logistic regression modelling was performed on hearing loss and aggregate variables to evaluate for any associations between symptoms and development of sensorineural or mixed hearing loss among EVD survivors (Fig. 3). Additional results, of variables not included in the model are available in Additional file 1: Table S3). The presence of any symptom affecting the middle or inner ear (p < 0.001), eye (p = 0.005), psychiatric (p = 0.019), and the central or peripheral nervous system (p = 0.037) were independently associated with significantly increased odds of hearing loss. Interestingly, the presence of any abnormal pulmonary physical exam findings or symptoms (p = 0.019) were associated with significantly decreased odds of hearing loss. After controlling for increasing age, all variables remained significant.

**Fig. 4:**
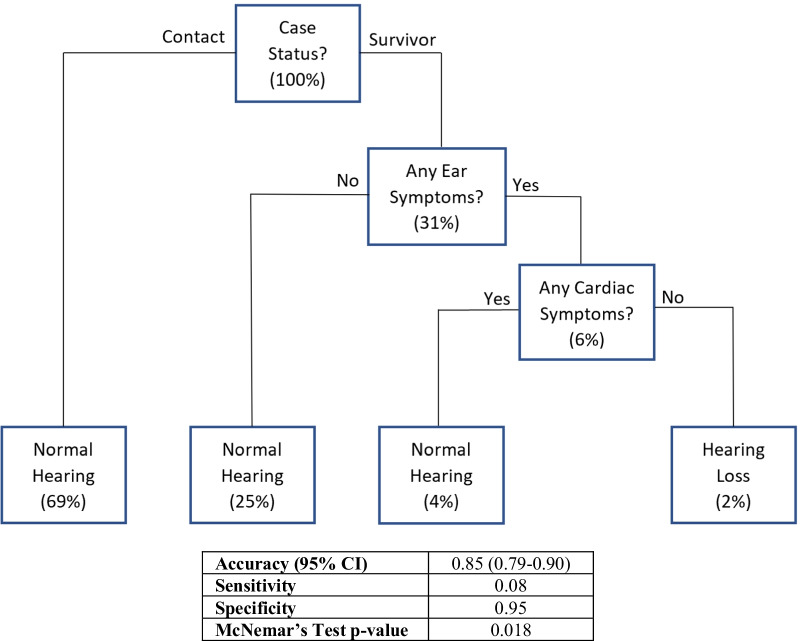
Decision tree model predicting sensorineural or mixed hearing loss among EVD cohort. Decision tree modelling produced the following algorithm to detect the most common form of hearing loss noted among EVD survivors and contacts. Prevalence of hearing loss at each node displayed in parentheses. Model characteristics presented in table underneath model

A classification decision tree was created in an attempt to determine non-linear relationships among variables predictive of hearing loss (Fig. 4). This model predicted hearing loss in EVD survivors with inner or middle ear symptoms without abnormal findings or symptoms affecting the heart. Overall this model had 86% accuracy (95% in predicting hearing loss, 12.5% sensitivity, 95% specificity, 30% positive predictive value, and 89% negative predictive value. The kappa value for this decision tree is 0.1146. However, this model was not significantly more accurate than the no information rate (accuracy = 88.12%, p = 0.01402). Additional modeling efforts attempted to improve upon this accuracy by limiting the cohort solely to EVD survivors (Additional file 1: Fig. S1). This EVD-Only model employed both Ear and Cardiac symptoms as similar data splits. In contrast, the EVD-Only model included MSK symptoms to further differentiate between survivors with cardiac symptoms. However, the EVD-Only model was not significantly different (p = 0.391) from the displayed model and was found to be less accurate in comparison to the displayed model and the no information rate (70%, no information rate = 73%, p = 0.774, kappa = 0.088).

## Discussion

Little is known regarding hearing loss in Post-EVD Syndrome. A literature review conducted in 2018 found that reports of hearing impairment in EVD survivors varied from 0% to as high as 66% [32, 33, 39–41]. However, only one of the studies included in this review included any objective measurement to assess for the presence of this symptom, which may leave many cases of hearing loss undetected [28]. The objective of this case-control study was to characterize and assess Post-Ebola Syndrome hearing loss and its relationship to other symptoms arising in the convalescent period. We found that 23% of EVD survivors had hearing loss as defined by pure tone averages of ≥ 25dB, nearly three times greater than the prevalence found among their household contacts. In addition, this study found that EVD survivors were significantly more likely to have complaints or abnormal physical exam findings affecting nearly every organ system. Among these reported complaints and findings, logistic regression modelling demonstrated that the development of any symptoms affecting the inner or middle ear, ophthalmologic, psychiatric, or nervous system significantly increased the risk of the development of hearing loss, while pulmonary symptoms significantly decreased risk of hearing loss among survivors.

Several viral diseases have been noted to cause hearing loss in the convalescent period. The more common viral etiologies include CMV, rubella, mumps, and tropical pathogens such as chikungunya, Zika, and Lassa fever viruses [16, 33, 42–49]. Although previous studies have suggested that direct viral damage to structures of the inner ear such as the stria vascularis, cochlea, and neuronal damage may be responsible in mumps and rubella, a host immune response to viral antigens in CMV, or the development of a vasculitis or autoimmune disease as in Lassa fever have been hypothesized [16, 45]. However, definitive evidence of any mechanism of pathogenesis is limited. Similarly, the mechanism through which Post-Ebola Syndrome hearing loss develops is still unknown. Previous studies have provided evidence of viral persistence in immune privileged sites such as the aqueous humor, semen, vaginal secretions, and the cerebrospinal fluid, as well as an association between this viral persistence and the development of convalescent symptoms [31, 50–54]. An additional study demonstrated that higher levels of viremia have been associated with the development of Post-Ebola Syndrome [55]. Collectively, these data indicate that a greater level of viremia may be required in order to penetrate immune privileged sites where delayed viral clearance and active replication may lead to greater amounts of direct viral damage to these protected structures. This hypothesis is in agreement with the results of the logistic regression modelling performed by this study which found that individuals who developed symptoms affecting the immune privileged sites of the eye or nervous system had 2.32 and 2.04 times the odds, respectively, of developing hearing loss. In contrast to the direct viral cytopathic effect, persistent immune activation has also been proposed as a possible mechanism partially responsible for the symptoms prevalent in Post-Ebola syndrome [56]. These two independent and competing processes of direct cytopathic effect and persistent immune activation may help to explain why the logistic regression model in this study found that individuals who developed pulmonary symptoms had significantly lower odds of developing an additional hearing impairment, as previous evidence has indicated that viral infections causing persistent immune activation can drive the development of a chronic lung disease [57].

To our knowledge, this is the largest systematic study of hearing loss among EVD survivors. The results of this study are unique in that they are the first to provide both air and bone conduction results in a cohort of EVD survivors and household contacts. These measurements found that 23% of EVD survivors had some form of hearing loss, which is significantly greater than their household contacts (9%). The most common type of hearing loss found among this survivor cohort was bilateral of either a mixed or sensorineural etiology. The majority of individuals with hearing loss were found to have mild hearing loss. Several previous studies have indicated that hearing loss is associated with an impaired ability to complete activities of daily living, increased rates of isolation, increased risk of depression, early cognitive decline in the elderly; increased risk of under- or unemployment, and lower socioeconomic status in adults; and increased risk of non-completion of secondary education, lower scores on IQ and verbal intelligence tests, and impaired language development in children [1, 58–64].

The relationship between hearing loss and decreases in economic output further emphasize the importance of providing treatment in these areas. Several previous studies have noted poverty as an important risk factor for infection and disease epidemics [65–69]. Specifically, poverty has been noted to be a powerful driver of EVD transmission throughout Sub-Saharan Africa [70–73]. However, attempts to provide hearing loss treatment in these areas will face significant challenges. The gold standard for diagnosis of hearing loss requires air and bone conduction audiometry, performed by a certified audiologist in a sound-proof room. None of these were available during the course of this study. However, this problem is common throughout Sub-Saharan Africa as previous data has indicated that there are less than 1 otologist per 100,000 individuals in the majority of the region [13, 74]. Although a number of validated mobile applications exist which may be used to screen for hearing loss, their cost may limit wide-spread adoption. The authors of this study sought to aid in this challenge by creating a decision tree algorithm to aid in hearing loss screening when proper conditions and technology were not available. Although the decision tree model was not significantly more accurate than the no information rate, its high specificity of 95% may prove useful when employed in conjunction with other validated high sensitivity instruments. The Five Minute Hearing test and the Hearing Handicap inventory have been found to have a combined sensitivity of 71% [75]. The design of an intervention implementing the use of these surveys and decision tree modelling in the future may serve as an important screening test to identify individuals in low-resource environments who would benefit from more intensive confirmatory testing and treatment. Further validations of designs such as these are necessary in order to demonstrate future reliability.

The results of this study are not without limitations. Significant differences were found between the age and gender distributions of the survivor and contact groups. This difference can be explained due to the higher incidence among women during the West African Ebola epidemic [76, 77]. This gender disparity was thought to be secondary to differences among gender roles in care-giving and funeral rites, increasing the risk of women in contracting EVD [76, 78, 79]. Age was also found to be significantly different among survivor and contact groups. This finding may be explained by a higher risk of exposure and contact with infected individuals among those of an increasing age [80, 81]. Additionally, the case-control design limits the ability to draw causal relationships. Given the study design and methodology data gathered from this cohort may not be generalizable to other EVD survivors and outbreaks. Recall bias may have been present, as the survey data collected was done so more than a year following the West African EVD outbreak. Additional study limitations include subjects lost to follow up, individuals with an incomplete set of data for analysis, and research team members with limited clinical experience related to otology and audiometry studies. Furthermore, in this study hearing loss was defined as ≥ 25 dB, since the time this study was conducted the WHO has redefined hearing loss to include individuals with PTA ≥ 20 dB. This discrepancy may lead to an underestimation of the prevalence of hearing loss in this population [4]. Despite these limitations, the results of this study are highly valuable, as they contribute to the growing understanding of viral disease sequelae and their importance. This study demonstrates the significant risk of hearing loss among EVD survivors, suggests possible mechanisms of pathogenesis, and provides a framework to identify EVD survivors with hearing loss in low resource environments.

## Conclusions

In conclusion, the results of this study demonstrate that 23% EVD survivors were found to have hearing loss following the resolution of the acute phase of illness. This hearing impairment was found to be significantly associated with the simultaneous development of symptoms affecting the eye and nervous systems. The association between ophthalmologic and neurologic symptoms and hearing loss may indicate a similar mechanism of pathogenesis. Future studies of EVD and Post-Ebola Syndrome would benefit from a more robust examination of the disease processes affecting the inner and middle ear in order to gain a fuller understanding of the spectrum of Post-Ebola Syndrome and to inform future interventions. The capacity to diagnose and provide treatment to affected individuals is severely limited in many tropical and low-income areas. Strategies designed to mitigate hearing loss in EVD survivors may also indirectly benefit other individuals affected by hearing loss.

## Supplementary Information

**Additional file 1:** **Table S1.** Ebola symptom questionnaire. **Table S2.** Ebola physical exam data. **Figure S1.** Decision tree modeling predicting sensorineural or mixed hearing loss among EVD survivors. **Table S3.** Odds ratios of logistic regression analysis of sensorineural or mixed hearing loss among EVD survivors.

## Data Availability

The datasets used and/or analysed during the current study available from the corresponding author on reasonable request.

## Abbreviations

EVD: Ebola Virus Disease
LMIC: Low and Middle Income Countries
WHO: World Health Organization
PTA: Pure tone average
CMV: Cytomegalovirus
SLESRC: Sierra Leone Ethics and Scientific Review Committee
