# Supplementary material for: The prevalence of Post-Ebola Syndrome hearing loss, Sierra Leone
Source: BMC Infect Dis. 2022 Jul 18;22:624. doi: 10.1186/s12879-022-07604-y (PMC9290210; doi:10.1186/s12879-022-07604-y)
Supplement: Supplementary file 1 — Additional file 1: Table S1. Ebola symptom questionnaire. Table S2. Ebola physical exam data. Figure S1. Decision tree modeling predicting sensorineural or mixed hearing loss among EVD survivors. Table S3. Odds ratios of logistic regression analysis of sensorineural or mixed hearing loss among EVD survivors. [file 12879_2022_7604_MOESM1_ESM.docx]

**Table S1. Ebola Symptom Questionnaire**

| **Variables  n (%)** | **Ebola Survivors  (n = 301)** | **Ebola Contacts  (n = 711)** | **p-value^*^** |
| --- | --- | --- | --- |
| **Systemic Symptoms and Complaints** | | | |
| **Fever** | 202 (67) | 304 (43) | <0.001 |
| **Excess Fatigue** | 72 (24) | 68 (10) | <0.001 |
| **Night Sweats** | 11 (4) | 11 (2) | 0.062 |
| **Unexplained Weight Loss** | 22 (7) | 13 (2) | <0.001 |
| **Hair Loss** | 12 (4) | 7 (1) | 0.003 |
| **Fainting** | 5 (2) | 5 (1) | 0.289 |
| **Loss of Appetite** | 46 (15) | 35 (5) | <0.001 |
| **Neurologic Symptoms and Complaints** | | | |
| **Headache** | 241 (80) | 426 (60) | <0.001 |
| **Paresthesia** | 29 (10) | 18 (3) | <0.001 |
| **Hallucinations** | 7 (2) | 1 (0) | 0.001 |
| **Psychiatric Symptoms and Complaints** | | | |
| **Difficulty Sleeping** | 34 (11) | 20 (3) | <0.001 |
| **Abnormal Behavior** | 27 (9) | 8 (1) | <0.001 |
| **Anxiety** | 12 (4) | 0 (0) | <0.001 |
| **Depression** | 14 (5) | 2 (0) | <0.001 |
| **Decreased Libido^†^** | 0 (0) | 0 (0) | NA |
| **Pharyngeal Symptoms and Complaints** | | | |
| **Hiccup** | 8 (3) | 6 (1) | 0.050 |
| **Sore Throat** | 29 (10) | 20 (3) | <0.001 |
| **Difficulty Speaking** | 6 (2) | 5 (1) | 0.139 |
| **Pulmonary Symptoms and Complaints** | | | |
| **Shortness of Breath** | 14 (5) | 8 (1) | 0.001 |
| **Cough** | 39 (13) | 65 (9) | 0.087 |
| **Cardiac Symptoms and Complaints** | | | |
| **Chest Pain** | 71 (24) | 72 (10) | <0.001 |
| **Heart Palpitations** | 36 (12) | 26 (4) | <0.001 |
| **Musculoskeletal Symptoms and Complaints** | | | |
| **Difficulty Walking** | 17 (6) | 11 (2) | 0.001 |
| **Joint Pain** | 136 (45) | 96 (14) | <0.001 |
| **Muscular Pain** | 60 (20) | 29 (4) | <0.001 |
| **Inner and Middle Ear Symptoms and Complaints** | | | |
| **Dizziness** | 26 (9) | 17 (2) | <0.001 |
| **Hearing Loss** | 23 (8) | 4 (1) | <0.001 |
| **Tinnitus** | 31 (10) | 11 (2) | <0.001 |
| **Ear Fullness** | 22 (7) | 7 (1) | <0.001 |
| **Gastrointestinal Symptoms and Complaints** | | | |
| **Nausea and Vomiting** | 26 (9) | 20 (3) | <0.001 |
| **Abdominal Pain** | 69 (23) | 90 (13) | <0.001 |
| **Diarrhea** | 24 (8) | 36 (5) | 0.100 |
| **Bloody Stool** | 13 (4) | 10 (1) | 0.009 |
| **Urorenal Symptoms and Complaints** | | | |
| **Increased Urination** | 17 (6) | 25 (4) | 0.167 |
| **Dermatologic Symptoms and Complaints** | | | |
| **Itchy Skin or Pruritus** | 38 (13) | 43 (6) | 0.001 |
| **Peeling Skin or Desquamation** | 9 (3) | 6 (1) | 0.022 |
| **Ophthalmologic Symptoms and Complaints** | | | |
| **Eye Dryness** | 7 (2) | 0 (0) | <0.001 |
| **Sensation of Burning Eyes** | 22 (7) | 12 (2) | <0.001 |
| **Vision Loss** | 11 (4) | 1 (0) | <0.001 |
| **Blurry Vision** | 64 (21) | 28 (4) | <0.001 |
| **Light Sensitivity or Photophobia** | 19 (6) | 8 (1) | <0.001 |
| **Itchy Eyes or Ocular Pruritus** | 84 (28) | 43 (6) | <0.001 |
| **Tearing or Epiphora** | 38 (13) | 28 (4) | <0.001 |
| **Eye Pain or Ophthalmalagia** | 37 (12) | 17 (2) | <0.001 |
| **Sensation of Foreign Bodies** | 22 (7) | 10 (1) | <0.001 |
| **Eye Redness or Conjunctivits** | 47 (16) | 19 (3) | <0.001 |
| **Increased "Eye Floaters" or Myodesopsia** | 10 (3) | 3 (0) | 0.001 |
| **Sensation of Flashing Light or Photopsia** | 15 (5) | 2 (0) | <0.001 |
| **Reproductive Symptoms and Complaints** | | | |
| **Missed Menstrual Cycle**^‡^ | 29 (10) | 39 (5) | 0.023 |
| **Heavy Menstrual Cycle**^‡^ | 12 (4) | 18 (3) | 0.296 |
| **Pregnancy**^‡^ | 34 (11) | 57 (8) | 0.122 |
| **Miscarriage**^‡^ | 0 (0) | 9 (1) | 0.111 |
| **Testicular Pain**^‡^ | 10 (3) | 6 (1) | 0.009 |
| **^*^Pearson’s Chi Squared Test; ^†^Questions asked of male subjects only; ^‡^Questions asked of female subjects only** | | | |

**Table S2. Ebola Physical Exam Data**

| **Findings**  **n (%)** | **Ebola Survivors**  **(n = 301)** | **Ebola Contacts**  **(n = 711)** | **p-value^*^** |
| --- | --- | --- | --- |
| **Systemic Exam Maneuvers and Vital Signs** | | | |
| **Height (Mean cm)** | 159 | 158 | 0.382 |
| **Weight (Mean kg)^†^** | 62 | 59 | 0.017 |
| **BMI (Mean BMI)^†^** | 25 | 24 | 0.013 |
| **Oxygen Saturation (Mean %)** | 96 | 98 | 0.418 |
| **Temperature (Mean C)** | 37 | 37 | 0.114 |
| **Heart Rate (Mean bpm) ^†^**^‡^ | 83 | 82 | 0.573 |
| **Respiratory Rate (Mean rpm) ^†^**^‡^ | 19 | 20 | 0.205 |
| **Systolic Blood Pressure (Mean mmHg) ^†^**^‡^ | 124 | 122 | 0.237 |
| **Diastolic Blood Pressure (Mean mmhg) ^†^**^‡^ | 79 | 76 | <0.001 |
| **Somnolent** | 1 (0) | 1 (0) | 1 |
| **Ill Appearing** | 3 (1) | 8 (1) | 1 |
| **Cachectic** | 0 (0) | 0 (0) | NA |
| **Ophthalmologic Exam Maneuvers** | | | |
| **EOMI** | 299 (99) | 708 (98) | 0.729 |
| **Abnormal Pupillary Response to Light** | 13 (4) | 12 (2) | 0.025 |
| **Icteric** | 3 (1) | 1 (0) | 0.151 |
| **Injected** | 1 (0) | 0 (0) | 0.658 |
| **Photophobia** | 0 (0) | 0 (0) | NA |
| **Other Ocular Findings** | 3 (1) | 0 (0) | 0.042 |
| **Pharyngeal Exam Maneuvers** | | | |
| **Cervical Lymphadenopathy** | 3 (1) | 4 (1) | 0.729 |
| **Pulmonary Exam Maneuvers** | | | |
| **Chest Wheezes on Auscultation** | 6 (2) | 16 (2) | 0.984 |
| **Chest Crackles on Auscultation** | 7 (2) | 11 (2) | 0.551 |
| **Chest Rhonchi on Auscultation** | 3 (1) | 7 (1) | 1 |
| **Chest Dullness on Percussion** | 0 (0) | 1 (0) | 1 |
| **Other Chest Findings** | 1 (0) | 1 (0) | 1 |
| **Cardiac Exam Maneuvers** | | | |
| **S1 Cardiac Sound Present** | 301 (100) | 709 (100) | 0.883 |
| **S2 Cardiac Sound Present** | 300 (100) | 706 (99) | 0.799 |
| **Normal Heart Rate** | 301 (100) | 707 (99) | 0.45 |
| **Regular Heart Rhythm** | 299 (99) | 705 (99) | 1 |
| **Heart Rub on Auscultation** | 0 (0) | 0 (0) | NA |
| **Heart Gallop on Auscultation** | 1 (0) | 0 (0) | 0.658 |
| **Heart Murmur on Auscultation** | 0 (0) | 0 (0) | NA |
| **Other Cardiac Finding** | 0 (0) | 1 (0) | 1 |
| **Gastrointestinal Exam Maneuvers** | | | |
| **Absent Bowel Sounds** | 0 (0) | 0 (0) | NA |
| **Abdomen Firm to Palpation** | 0 (0) | 0 (0) | NA |
| **Mass Palpated** | 0 (0) | 0 (0) | NA |
| **Abdomen Tender to Palpation** | 13 (4) | 11 (2) | 0.015 |
| **Epigastric Tenderness** | 9 (3) | 1 (0) | 0 |
| **Right Upper Quadrant Tenderness** | 0 (0) | 2 (0) | 0.883 |
| **Right Lower Quadrant Tenderness** | 2 (1) | 1 (0) | 0.442 |
| **Left Upper Quadrant Tenderness** | 0 (0) | 0 (0) | NA |
| **Left Lower Quadrant Tenderness** | 2 (1) | 2 (0) | 0.734 |
| **Hepatomegaly Present** | 9 (3) | 2 (0) | 0.001 |
| **Splenomegaly Present** | 49 (16) | 49 (7) | <0.001 |
| **Other Abdominal Finding** | 9 (3) | 10 (1) | 0.149 |
| **Musculoskeletal Exam Maneuvers** | | | |
| **Extremities Well Perfused** | 7 (2) | 14 (2) | 0.902 |
| **Distal Pulse Grade (Mean ± SD)** ^‡^ | 2 | 2 | 0.1083 |
| **Lower Extremity Edema Present** | 1 (1) | 3(0) | 1 |
| **Other Extremity Finding** | 0 (0) | 1 (0) | 1 |
| **Decreased Range of Motion to Any Joint** | 12 (4) | 8 (1) | 0.006 |
| **Joint Effusion Present** | 0 (0) | 0 (0) | NA |
| **Any Musculoskeletal Tenderness** | 12 (4) | 0 (0) | <0.001 |
| **Neurologic Exam Maneuvers** | | | |
| **Abnormal Gait** | 0 (0) | 0 (0) | NA |
| **Abnormal Muscle Tone** | 0 (0) | 0 (0) | NA |
| **Abnormal Romberg Test** | 0 (0) | 0 (0) | NA |
| **Abnormal Heel to Shin Test** | 0 (0) | 0 (0) | NA |
| **Abnormal Finger to Nose Test** | 0(0) | 0 (0) | NA |
| **Abnormal Knee Pat Test** | 0 (0) | 0 (0) | NA |
| **Abnormal Heel Walk** | 0 (0) | 0 (0) | NA |
| Abbreviations: BMI, Body Mass Index; EOMI, Extra Ocular Muscles Intact; PERRLA, Pupils Equally Round and Reactive to Light and Accommodates; ^*^Pearson’s Chi-Squared Test; ^†^Result Only Includes Individuals Aged ≥ 15 Years; ^‡^Student’s T-Test | | | |

**Figure S1. Decision Tree Modeling Predicting Sensorineural or Mixed Hearing Loss Among EVD Survivors**


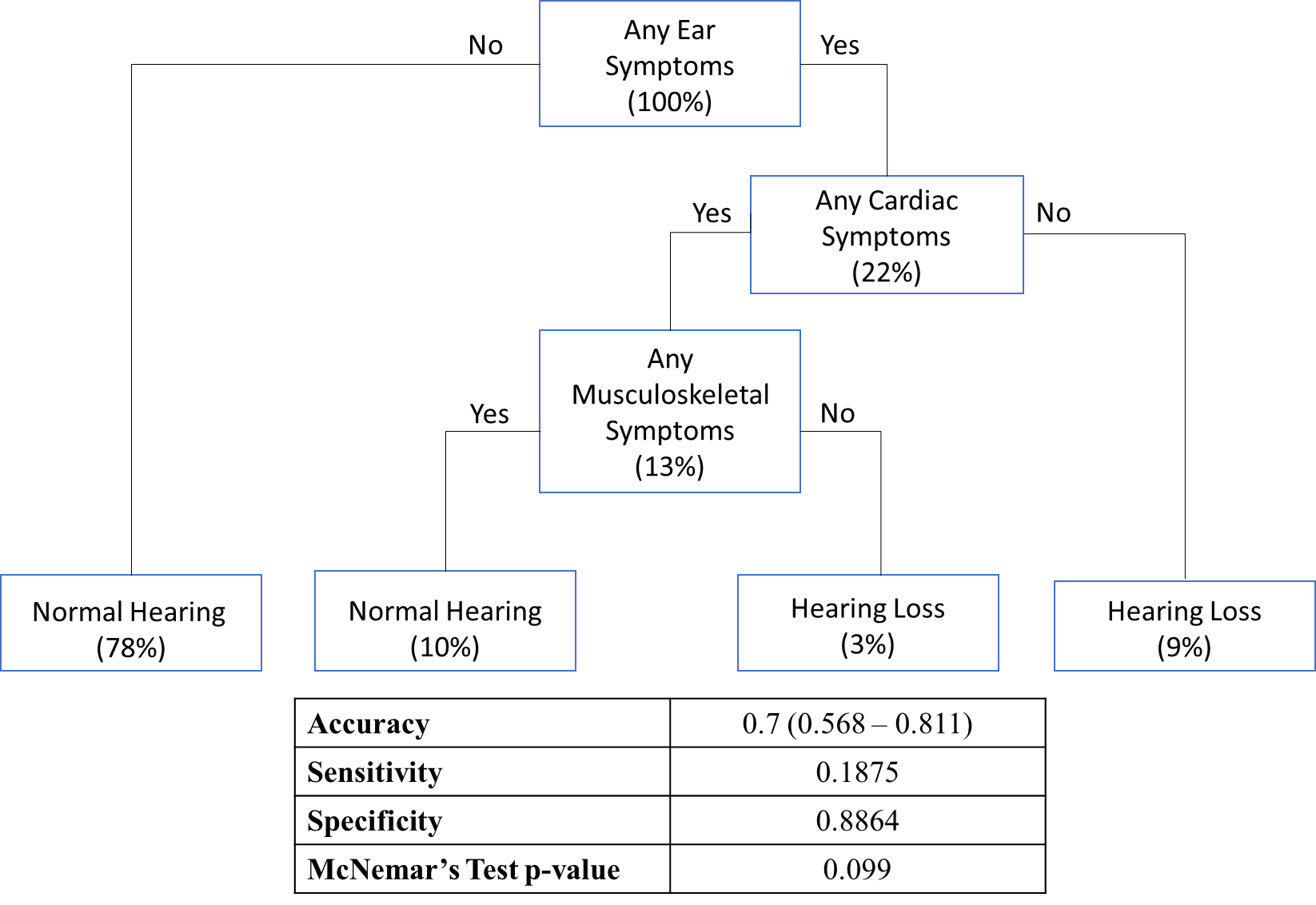


Decision tree modelling produced the following algorithm to detect the most common form of hearing loss noted among EVD survivors. Prevalence of hearing loss at each node displayed in parentheses. Model characteristics presented in table underneath model.

**Table S3. Odds Ratios of Logistic Regression Analysis of Sensorineural or Mixed Hearing Loss Among EVD Survivors**

| **Aggregate Variable** | **Odds Ratio (95% CI)** | **p-value prior to control** | **p-value after control** |
| --- | --- | --- | --- |
| **Inner and Middle Ear** | 3.79 (1.98 - 7.08) | <0.001 | <0.001 |
| **Ophthalmologic** | 2.32 (1.28 - 4.13) | 0.004 | 0.019 |
| **Psychiatric** | 2.3 (1.12 - 4.53) | 0.019 | 0.031 |
| **Neurologic** | 2.04 (1.08 - 4.16) | 0.034 | 0.040 |
| **Pulmonary** | 0.37 (0.15 - 0.81) | 0.019 | 0.021 |
| **Musculoskeletal** | 1.374 (0.75 - 2.471) | 0.295 | 0.540 |
| **Cardiac** | 1.3 (0.673 - 2.421) | 0.422 | 0.482 |
| **UroRenal** | 1.214 (0.402 - 3.186) | 0.710 | 0.723 |
| **Pharyngeal** | 1.053 (0.426 - 2.36) | 0.905 | 0.712 |
| **Reproductive** | 1.037 (0.578 - 1.799) | 0.900 | 0.651 |
| **Gastrointestinal** | 1.032 (0.557 - 1.85) | 0.917 | 0.597 |
| **Systemic** | 0.892 (0.306 - 3.797) | 0.854 | 0.735 |
| **Dermatologic** | 0.822 (0.358 - 1.725) | 0.623 | 0.729 |
| p-values are displayed prior to and after control of age | | | |
